# Optimizing intact skull intrinsic signal imaging for subsequent targeted electrophysiology across mouse visual cortex

**DOI:** 10.1038/s41598-022-05932-2

**Published:** 2022-02-08

**Authors:** Armel Nsiangani, Joseph Del Rosario, Alan C. Yeh, Donghoon Shin, Shea Wells, Tidhar Lev-Ari, Brice Williams, Bilal Haider

**Affiliations:** 1 Biomedical Engineering, Georgia Institute of Technology & Emory University, Atlanta, USA; 2 Electrical and Computer Engineering, Georgia Institute of Technology, Atlanta, USA; 3 Biology & Computer Science, Georgia State University, Atlanta, USA

**Keywords:** Neuroscience, Visual system, Extrastriate cortex, Striate cortex, Optical imaging, Electrophysiology

## Abstract

Understanding brain function requires repeatable measurements of neural activity across multiple scales and multiple brain areas. In mice, large scale cortical neural activity evokes hemodynamic changes readily observable with intrinsic signal imaging (ISI). Pairing ISI with visual stimulation allows identification of primary visual cortex (V1) and higher visual areas (HVAs), typically through cranial windows that thin or remove the skull. These procedures can diminish long-term mechanical and physiological stability required for delicate electrophysiological measurements made weeks to months after imaging (e.g., in subjects undergoing behavioral training). Here, we optimized and directly validated an intact skull ISI system in mice. We first assessed how imaging quality and duration affect reliability of retinotopic maps in V1 and HVAs. We then verified ISI map retinotopy in V1 and HVAs with targeted, multi-site electrophysiology several weeks after imaging. Reliable ISI maps of V1 and multiple HVAs emerged with ~ 60 trials of imaging (65 ± 6 min), and these showed strong correlation to local field potential (LFP) retinotopy in superficial cortical layers (r^2^ = 0.74–0.82). This system is thus well-suited for targeted, multi-area electrophysiology weeks to months after imaging. We provide detailed instructions and code for other researchers to implement this system.

## Introduction

The mouse has become an important tool for investigation of the mammalian visual cortex. Anatomical studies in mice reveal strong interconnections of primary visual cortex (V1) with multiple higher visual areas (HVAs)^1–3^; this hierarchical cortical organization parallels that of the primate visual system^4^. Moreover, V1 and HVAs in mice show retinotopic organization, such that visual space maps topographically to cortical space. Measuring cortical responses to retinotopic visual stimulation with intrinsic signal imaging (ISI) of hemodynamics allows functional localization of V1 and HVAs in the intact mouse brain^5,6^. However, cortical hemodynamic responses show small amplitude changes (< 1% relative to ongoing fluctuations^7^), so investigators typically thin or remove the skull (and often the dura) of adult mice for maximum signal quality in V1 and HVAs^2,5,7^; further, ISI is typically performed during anesthesia, where controlled conditions permit large numbers of stimulus repetitions and averaging that overcomes small signal amplitudes and background fluctuations^5,8,9^.

These well-established ISI methods pose some limitations for subsequent electrophysiological recordings. First, skull removal or thinning can lead to inflammation, bone remodelling, scar formation, and neural plasticity in as little as a week^5,10–12^, constraining the timeframe of subsequent electrophysiological measurements. Optimizing a transcranial ISI system would preserve skull integrity and ensure optimum mechanical stability and physiological conditions during delicate electrophysiological recordings, particularly multi-site silicon probe or patch clamp recordings^13,14^. Second, most ISI protocols utilize anesthesia and sedation, where hundreds of visual stimulus repetitions and hours of imaging are needed for high-resolution retinotopic maps^5,8,9,15^. Prolonged anesthesia, even at low concentrations, can induce lasting effects on visual task performance in rodents^16,17^, potentially impairing subjects undergoing behavioral training that lasts weeks to months^18,19^. Quantifying the minimum amount of data necessary for intact skull ISI would help mitigate any unneeded consequences of prolonged or repeated anesthesia. To our knowledge, no study has optimized ISI for intact skull conditions in adult mice, quantified the minimum sample size (imaging duration) necessary for reliable and repeatable maps of V1 and HVAs, then verified these retinotopic maps directly with electrophysiological measurements.

Here, we optimized and validated performance of an intact skull ISI system for mice. We instilled several quality control checkpoints to ensure robust detection of transcranial ISI signals. We quantified the duration of imaging necessary for reliable retinotopic maps of V1 and HVAs, and then verified these ISI maps with targeted awake and anesthetized electrophysiological recordings, often several weeks after the initial imaging. Our findings reveal a high degree of correspondence between ISI map retinotopy and direct electrophysiological measurements in superficial cortical layers. Our system specifications, protocol, and codebase are all made publicly available, providing a useful tool for investigators wishing to pair minimally invasive transcranial ISI with subsequent targeted electrophysiology in the mouse visual system.

## Results

### Strategy for optimal acquisition of intact skull intrinsic signal imaging

**Figure 1:**
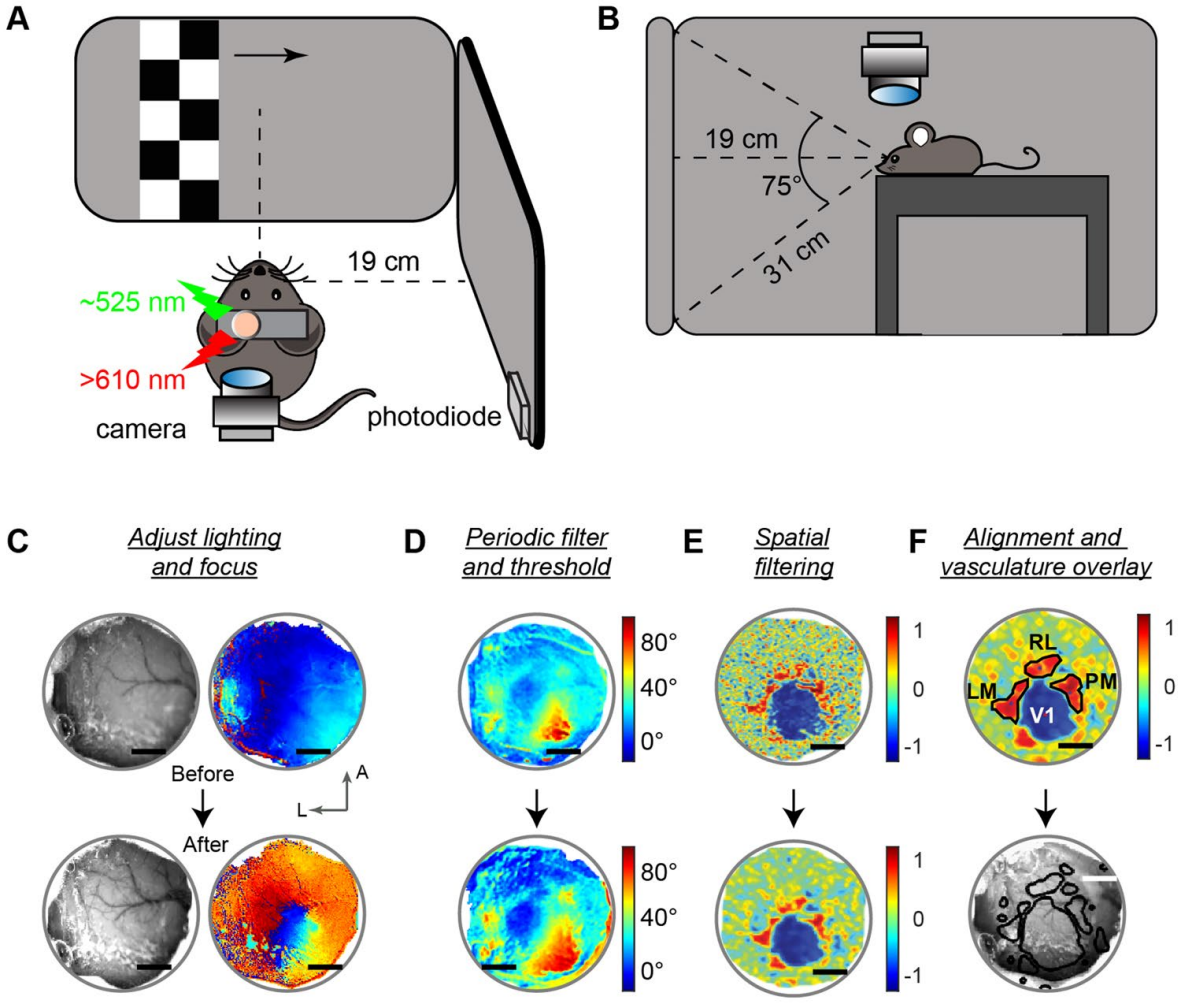
Experimental setup and optimization steps for transcranial intrinsic signal imaging. (**A**, **B**) Position of mouse and monitors displaying visual stimuli. Eyes were vertically and horizontally centered at each monitor (~ 19 cm away from each), and these formed a right angle. A primary computer controlled the main system components: light intensity (Red light: λ =  > 610 nm; Green light: λ =  ~ 525 nm); a complementary metal–oxide–semiconductor (CMOS) camera coupled to a tandem lens macroscope; and a photodiode recording the timing of visual display events. A secondary computer displays visual stimuli and communicates via UDP with the primary computer. The visual stimulus is a contrast-reversing (6 Hz) checkerboard pattern drifting across the screen (0.055 Hz). Stimuli drifted from left to right (and right to left) to map retinotopy in azimuth and drifted from bottom to top (and top to bottom) to map elevation. (**C**) Experiments start with a coarse test for hemodynamic signals under green light (see Table 1, “Methods” section), then move to acquisition with red light. If green light imaging failed to generate high quality signals (top, before), the illumination and focal plane was adjusted (bottom). Color scale shows normalized signal intensity and does not correspond to visual space. Scale bar = 1 mm. (**D**) After signal optimization, red light imaging commences. Algorithms exclude noisy frames based on a minimum signal to noise (SNR) threshold for periodic responses at the stimulus frequency (see “Methods” section), resulting in higher quality maps (bottom). Resulting absolute phase maps are shown (− 10° to 120° in azimuth). (**E**) Optimal spatial filtering of high SNR frames defines clear areal boundaries in visual field sign maps (see Fig. 2C and Fig. S4C–F). Visual field sign (VFS) maps shown, scale from − 1 (sign negative areas) to 1 (sign positive areas). (**F**) Retinotopic maps, VFS maps and area contours (top) are aligned to vasculature images (bottom, acquired with green light) for registration of areas with visible vasculature landmarks. Investigators assess alignment, coverage range, size, and location of areas relative to expected^6^. See also Table 2. (**C**–**F**) All in same mouse (Mouse 1; Fig. S3).

Our ISI protocol is inspired by prior work^2,5^, but provides two major improvements tailored for ISI integration with subsequent multi-site electrophysiology. First, we have optimized and validated imaging signal quality using a modified intact skull cranial window preparation (Fig. 1). Unlike skull thinning or removal, which can cause inflammation, scarring, or bone regrowth in as little as 7 days^5,20^, our method allows for less invasive pre-imaging preparation, preserving the skull integrity for high-quality electrophysiological recordings with multiple sequential craniotomies. The optimal combinations of headplates, glass coverslips, and adhesives were determined after a series of pilot experiments. We tested different coverslip thicknesses (0.09–0.12 mm, 0.13–0.17 mm) and diameters (e.g., 3 or 5 mm). We selected coverslips that provided the best balance of spatial coverage, qualitative optical clarity (sharpness of vasculature immediately after glue polymerization), and minimal amount of glue (and waiting time) needed for stable polymerization. The 5 mm diameter coverslip with 0.09–012 mm thickness and Vetbond provided the optimal combination of clarity, cortical spatial coverage, and ease of implantation. Thicker coverslips required too much glue and/or time for polymerization, and Vetbond provided the greatest clarity of vasculature as compared to an alternate adhesive (CA Glue). Further, Vetbond plus a coverslip provided long-lasting clarity and smoothly graded retinotopic maps as compared to glue alone with no coverslip (Fig. S2C). This is likely due to the coverslip providing a barrier from mechanical degradation and air exposure that increases opacity over time. Once these window parameters were optimized, we tested various headplates with different sized chambers to assess mechanical stability and ease of access during electrophysiology experiments. We found that the 5 mm coverslip bonded inside a headplate with an 11 mm circular opening (Fig. S2) provided a highly repeatable and mechanically stable platform for ISI, subsequent cranial window removal, and multiple sessions of mechanically stable high-quality electrophysiology recordings.

Our cranial windows provided good quality imaging several weeks after implantation. Cranial windows and ISI maps are shown for all individual mice (Fig. S3; n = 10 mice imaged from 1 to 6 weeks after implantation). We observed strong correlations between first day and subsequent day maps (average 15.5 days later), both relative to a fixed reference map within mouse (Fig. S3A; Pearson r = 0.82 ± 0.07 across all imaging days; n = 4 mice with long term imaging). The general steps of the imaging protocol and references to all MATLAB code required for image acquisition, processing, and troubleshooting are described in Table 1.

**Table 1:** ISI protocol.

Experiment stages	Step #	Time (min)	Software used	Description
Evoked signal detection (Fig. 1C)	1	5		Anesthesia, sedation, camera placement
	2	10	primary_script.m secondary_script.m	The camera is focused to the cortex and acquires images of vasculature. A test experiment (green filter and illumination) with a single block of visual stimulus presentation is used to detect global hemodynamic signal
			Matrox Intellicam	
Prep for retinotopic map acquisition	3	0.5	primary_script.m; secondary_script.m	Adjust focus to intracortical plane (~ 0.1–0.5 mm below cranial surface)
	4	0.5		Switch to red filter and illumination
Azimuth map acquisition	5	30.5–61	primary_script.m; secondary_script.m	Load *horizontal.param* in *primary_script.*m and run script for visual stimulation in azimuth
Elevation map acquisition	6	30.5–61		Load *vertical.param* in *primary_script.*m and run script for visual stimulation in elevation
Retinotopic map analysis and display (Fig. 1F; Sup. Fig. S3)	7	10–20	run_first.m	Load *Post-imaging* folder and run *run_first.m* to construct retinotopic and VFS maps. Processing includes:
				– Retrieval of slow fluctuations in intensity across all imaging frames (frequency domain)
				– Azimuth and elevation phase maps for each trial
				– SNR thresholding
				– Average azimuth and elevation retinotopic maps per session
				– Combination of maps from multiple imaging sessions
				– Overlay of retinotopic, VFS, vasculature
Alignment of craniotomies to retinotopic contours (Fig. 4B)	8	5	Align.m	Load *Post-imaging* folder and run *Align.m* to align craniotomy images to retinotopic contours

We also added online processing to our system to facilitate troubleshooting during the experiment. Before experimenters commit to multiple repeated blocks of red light imaging, green light images were acquired and rapidly analyzed (with temporal compression) to provide feedback of signal quality to the user (Fig. 1C). Although green light imaging captures spatially coarse signals due to changes in blood volume, vasculature dilation, and capillary blood recruitment in addition to cortical activity^21^, the higher SNR of green versus red light signals provides a robust estimate of overall hemodynamic signals in few trials. If coarse hemodynamic signals appear poor, users can immediately proceed to common troubleshooting measures (detailed in Table 2).

**Table 2:** Troubleshooting.

Case	Intervention	Comment
Failure to detect signal (Table 1, steps 1–2)	a. Adjust camera focus deeper	a. Focusing the camera at inappropriate level (e.g. at the dural surface) reduces signal amplitude (see “Methods”, “Imaging procedures” sections)
	b. Adjust camera position	b. Cranial window must be positioned at the center of the camera, reflecting the brightest light
	c. Decrease/increase light intensity	c. Hemodynamic signals evoke small reflectance intensity changes. Too little light may prevent signal detection
	d. Adjust anesthesia level	d. High levels of anesthesia impair visual signals. It is important to pair sedation with minimal maintenance anesthesia during imaging
Software or Image acquisition freezes/crashes	a. Clear all or Restart MATLAB	a. Clear workspace or restart MATLAB to flush memory
	b. Unplug/plug data acquisition (DAQ) device	b. DAQ device may power off if software freezes, preventing additional recordings
	c. Reboot primary computer	c. Extreme case, only when the experimenter is unable to acquire images

**Figure 2:**
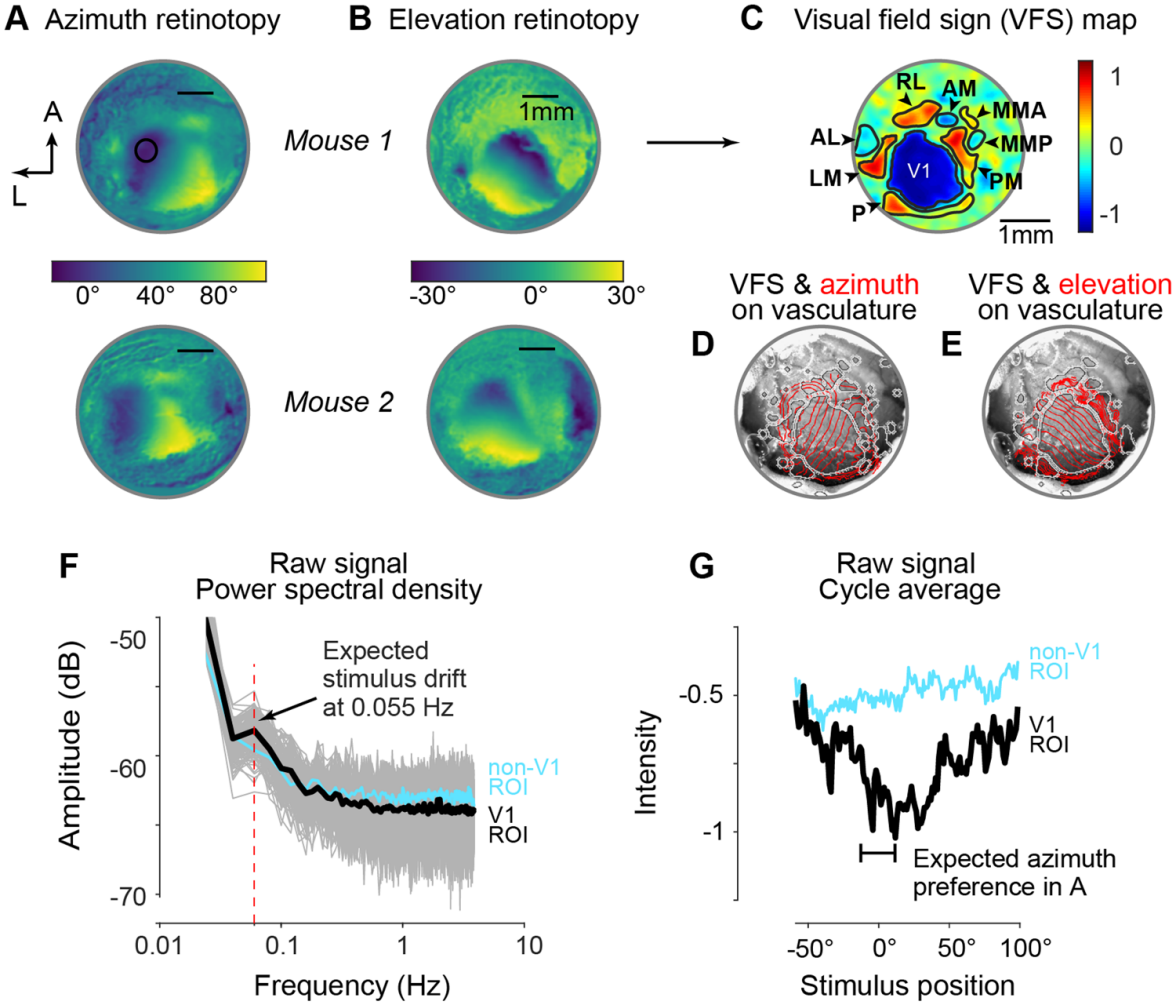
Retinotopic maps of azimuth, elevation, and visual field sign with intact skull ISI. (**A**) Retinotopic maps in azimuth from 2 mice (Mouse 1 and 2; see Fig. S3) with 5-mm intact skull cranial windows. Colorbar shows spatial location of visual stimulus driving maximal Fourier response at each image pixel (see “Methods” section). Scale bar is 1 mm. (**B**) As in (**A**), for elevation maps in same mice. (**C**) Visual field sign (VFS) map for mouse 1 showing cortical area boundaries, computed from azimuth and elevation maps. Scale is − 1 to 1 (see “Methods” section for calculation). Identified areas V1 (primary visual cortex), Area P (posterior aspect of visual cortex), LM (lateromedial), AL (anterolateral), RL (rostrolateral), AM (anteriomedial), PM (posteromedial), MMA (Medio-medial-anterior), and MMP (Medio-medial-posterior). Identified areas correspond to prior reports with excised skull cranial windows^6^. (**D**) Overlay of VFS (white) and azimuth (red) retinotopic map contours (10° increments) on image of vasculature of Mouse 1. Custom processing in finalized software package allows users to define visual areas from VFS map, and automatically align maps to vasculature. (**E**) As (**D**), for elevation map contours. (**F**) Power spectrum of raw reflectance from multiple pixels (black circle in **A**) across 2880 imaging frames and 16 stimulus trials (grey traces). A peak is present in the average response (black) at the frequency of visual stimulus (0.055 Hz, red dashed line). Blue trace shows power spectrum of pixels in adjacent non-visual cortical area from same trials. (**G**) Average intensity versus stimulus position averaged across 200 stimulus cycles. Maximum intensity change (decreased reflectance) occurs near preferred stimulus location expected from azimuth map (black circle in **A**). Maximum slightly displaced due to hemodynamic delay for stimulus drifting from negative to positive azimuth (Fig. 1A). Blue trace shows cycle average for pixels outside of visual areas.

To further increase signal quality control, we added an algorithm to detect and discard individual trials with poor signals that degrade average retinotopic map quality (Fig. 1D). This step is consistent with previous studies showing that noisy frames degrade imaging results^22^. The algorithm first extracted the Fourier phase and amplitude of the signal at the stimulus drift frequency for each trial of red imaging (Fig. 2F). The normalized variance of each trial phase map was then computed. Phase maps with low variance were found to consist mostly of noisy trials without stimulus-driven pixel changes in expected ROIs. We found that a threshold for discarding frames with normalized variance < 0.6 provided the best results across all experiments. To increase user control of this process, the code displays averaged phase maps from each block of trials at different variance thresholds, and the user is free to adjust this. On average ~ 10% of trials per session were discarded due to noisy image data, and these tended to cluster at the beginning of imaging sessions, when anesthesia level transitioned from induction to maintenance. This suggests a main source of noise arose from anesthetic depth and effects on neurovascular coupling^7^. These noise-reduction procedures likely improved detection of HVAs in fewer trials, since HVAs are smaller than V1 and more sensitive to small changes in signal quality.

We also quantified the effect of smoothing parameters on map reliability in V1 and HVAs. We determined the optimal amount of 2-D Gaussian spatial filtering that improves the clarity and sharpness of retinotopic maps and visual area borders (Fig. 1E), without distorting ground-truth retinotopic coordinates. We swept through 6 spatial filtering parameters and computed the correlation between smoothed areal maps and a (fixed) reference map. Areal reliability of V1 and HVAs was significantly impacted with parameters lower or greater than the optimal Gaussian kernel width (found to be σ = 3–5 pixels, equivalent to 18–30 µm, similar to prior studies^4,7,8^); similarly, the smoothness and steepness of borders between V1 and HVAs was also compromised when using non-optimal smoothing parameters.

Finally, we implemented an algorithm for improved and automatic alignment of image frames across sessions and days from the same subjects (Fig. S1D). This enables investigators to perform multiple short imaging sessions across multiple days and align all image frames to produce an averaged map across sessions. This reduces the need for prolonged anesthetized experiments, minimizing physiological stress associated with recovery from anesthesia^23^, which could be particularly beneficial in subjects undergoing concurrent behavioral training in challenging tasks^18,24,25^.

These modified procedures enable construction of high-quality transcranial retinotopic maps, comparable in quality to previous studies where the cranium is thinned or removed (including dura removal in some cases)^2,5,7^. Typical visual field coverage spanned − 30° to 30° in altitude, and − 10° to 120° in azimuth (Fig. 2A). A frequency domain analysis of reflectance from V1 in representative retinotopic maps showed a clear peak at the drift frequency of the visual stimulus (Fig. 2F), and a sharp change in reflected light intensity at the expected spatial location (Fig. 2G; slightly displaced due to expected hemodynamic signal lag), and no such changes in adjacent non-visual cortical regions. Our protocol and code also provide semi-automated alignment of the retinotopic and visual field sign maps to the vasculature observed through the cranial window (Fig. 2D,E; Fig. S2D). This step is important because it allows investigators to align the ISI retinotopic maps and VFS maps to visible vasculature landmarks that guide selection of sites for craniotomies.

### Data length requirements for reliable and repeatable retinotopic maps

**Figure 3:**
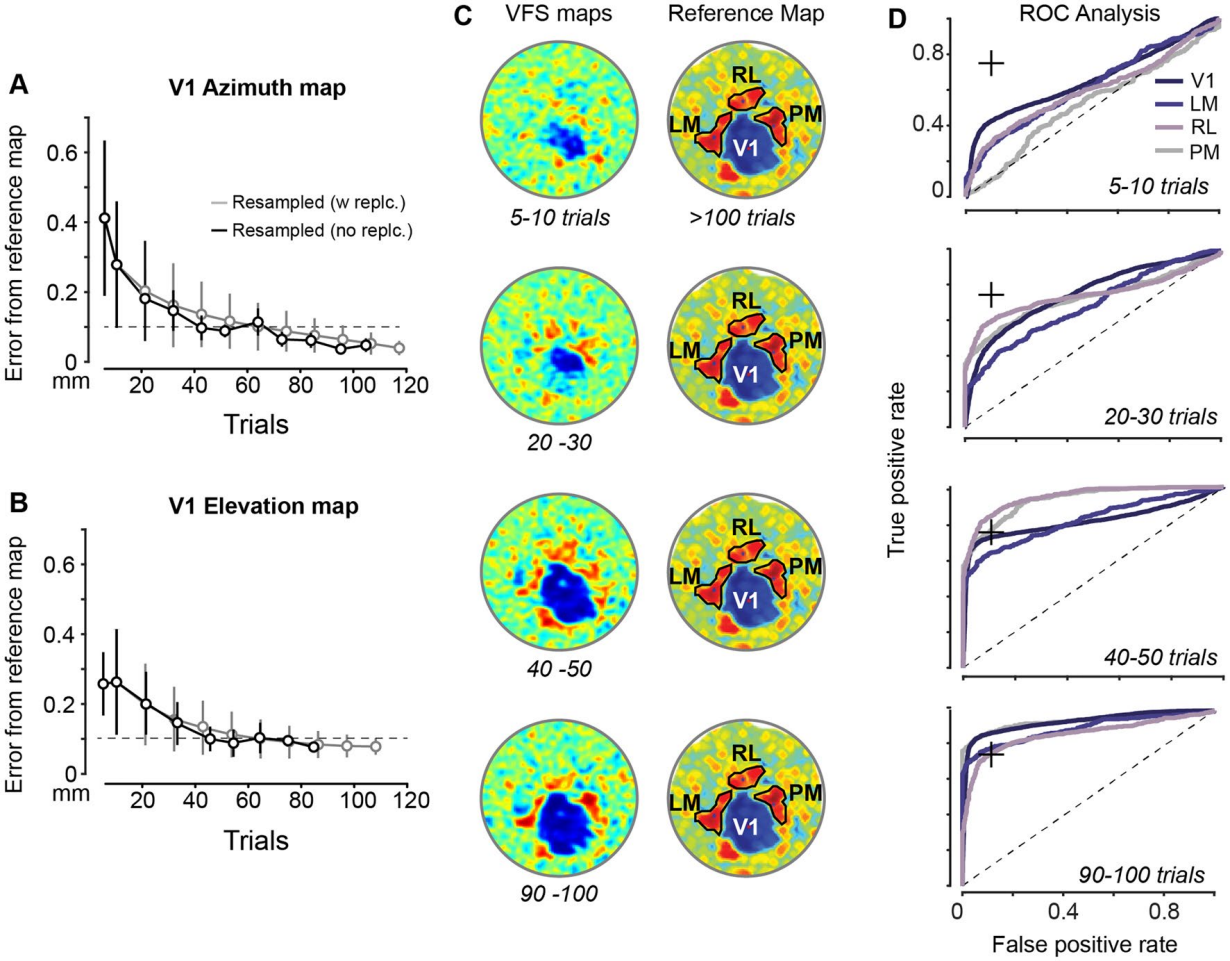
Quantifying resolvability of V1 and HVAs as a function of trials. (**A**) Error of estimated azimuth receptive field (RF) locations in V1 as a function of number of trials. Retinotopic contour maps computed from varying trial numbers (abscissa), and error estimated as Euclidean distance of RF locations in trial-limited maps versus reference map (mean distance ± SD; centroids of contours binned at 10°, Fig. 2D). Reference map computed from 190 trials (5 recording sessions), trial-limited maps computed by randomly subsampling from these 190 trials with (grey) or without (black) replacement. Error in estimated RF locations falls < 0.1 mm (dashed line) within 60 trials. No significant difference in centroids of azimuth contours for reference maps and resampled maps with > 60 trials (see Results). (**B**) Same mouse as (**A**), for elevation RFs. Reference maps from 170 trials (5 recording sessions). No significant difference in centroids of elevation contours (e.g., Fig. 2E) between reference maps and resampled maps with > 60 trials (*p* = *0.325*, Mann–Whitney U with Bonferroni correction). (**C**) Trial limited VFS maps (left) versus reference VFS map (right). Same sessions as (**A**,**B**). Reference map shows contours for V1 and only 3 HVAs for clarity: LM (lateromedial area), RL (rostrolateral), and PM (posteromedial). (**D**) Receiver operating characteristic (ROC) curves computed for centroid detection of V1 and 3 sign positive HVAs (LM, RL, PM) for same trial limited VFS maps in (**C**). True positives evaluated as average pixel values in areal contours defined from reference VFS maps versus noise areas outside of visual cortex (Fig. S2). Areal boundaries for V1, RL, and PM pass detection threshold (75% accuracy, + symbol) after > 50 visual stimulus trials. LM areal boundaries pass threshold after > 70 trials. Sign negative areas AL and AM were also readily identified with > 70 trials (e.g., Fig. 2C). (**A**–**D**) All from Mouse 1 (Fig. S3).

We next determined the optimal amount of data needed to generate reliable and repeatable retinotopic maps in our conditions. Prior work with thin skull or excised skull cranial windows suggests that ~ 100 sweeps of visual stimuli are required for adequate retinotopic and VFS maps; however, to the best of our knowledge, these suggested sample sizes lack clear quantitative justification^5,8^. Further, it was also possible that transcranial ISI would require significantly more data to reliably resolve signals from V1 and HVAs. Therefore, we first determined the amount of data necessary to construct well-defined retinotopic maps. Our general strategy was to compute retinotopic maps using different subsampled amounts of trials and to compare these to an overall ‘reference’ retinotopic map constructed from all eligible trials across all imaging sessions within subject (> 190 trials over several days; see “Methods” section). We measured the centers (centroid) and extent (boundaries) of each identified cortical area defined by the VFS reference maps. We then plotted the average Euclidian distance (error) between V1 centroids derived from the various subsampled maps and the reference map. We found that the average error fell below 100 μm after ~ 60 visual stimulus trials were included for map generation (Fig. 3A,B). We defined an error of < 100 μm acceptable since this is the average size of craniotomies made for silicon probe recordings. We found no significant difference between V1 centroids in the reference map versus subsampled maps constructed from > 60 visual stimulus trials (*p* > *0.05*, Mann–Whitney U with Bonferroni correction). This minimum trial number was established from one mouse (Fig. 3) then tested and confirmed with 4 other mice (Supplementary Fig. S4B; Average error of 80 ± 16 μm after 69 ± 5 trials, mean ± SD).

We next determined the amount of data needed to estimate the boundaries of V1. To do this, we used the reference VFS map to define the extent of V1 (Fig. 3C; reference map constructed from > 190 single trial azimuth and elevation maps; see Methods). We then used the same subsampling strategy to determine the minimum number of trials necessary for resolution of V1 from the background signal. All pixels within V1 in the reference VFS maps were considered “signal”. We then defined a “noise” region outside of V1 that also did not contain any HVA (Supplementary Fig. S2D). The separability of the signal and noise regions were compared in the various subsampled versus reference VFS maps to define the minimum amount of data needed to define the extent of V1. The analysis revealed that a clear separation between V1 signal versus noise distributions starts when > 43 visual stimulus trials were used to construct a VFS map (Fig. S4A). This finding was consistent with the visual inspection of VFS maps (Fig. 3C).

We next used this same procedure to identify the minimum number of trials needed to resolve multiple HVAs. ROC analysis was performed to determine whether pixel intensity (signal) in a visual cortical region of interest (ROI) is distinguishable from intensity in an adjacent non-visual cortical region (noise). Visual cortical ROIs were determined using the reference map constructed from all trials, and an adjacent non-visual ROI defined the noise distribution (Fig. S2D). A classification boundary was determined and then applied to ROIs in maps constructed from subsampled data with increasing numbers of trials. The curve plotting the ratio of true positives to false positives determines the accuracy of the classification boundary, with an area under the receiver operating characteristic curve (AUROC) of 0.5 (diagonal line in Fig. 3D) equal to chance level classification. We then evaluated classification performance in the visual vs non-visual ROIs from subsampled VFS maps constructed with increasing numbers of trials.

We generated receiver operating characteristic (ROC) curves for primary visual cortex (V1), and for lateromedial area (LM), rostrolateral area (RL), and posteromedial area (PM) to assess our ability to detect these HVAs from noise as the number of trials increases. For this analysis, the size and extent of each area was determined from a reference VFS map. Then, using similar resampling methods as described previously, multiple subsampled VFS maps were created by aggregating different numbers of visual stimulus trials (Fig. 3C). The area under the ROC curves (AUROC) comparing the pixel intensity inside and outside these visual areas shows that the full extents of visual areas PM and RL were detectable at 75% accuracy level after ~ 54 trials (Fig. 3D), while area LM necessitated ~ 75 trials. Similar trial duration criteria were found even when we did not constrain the pixel area by the VFS reference map, but instead analyzed the pixel SNR at centroids of sign positive (or negative) areas detected in maps generated with increasing numbers of trials. Although we only quantified detectability of areas LM, RL, PM, the reference maps also show that areas P, AL, AM, and several others were clearly resolvable after 75 trials (see Fig. 2C). These other areas are not analyzed in detail here since they were not extensively targeted for electrophysiology, discussed next.

### Validating intrinsic signal imaging with targeted electrophysiology

**Figure 4:**
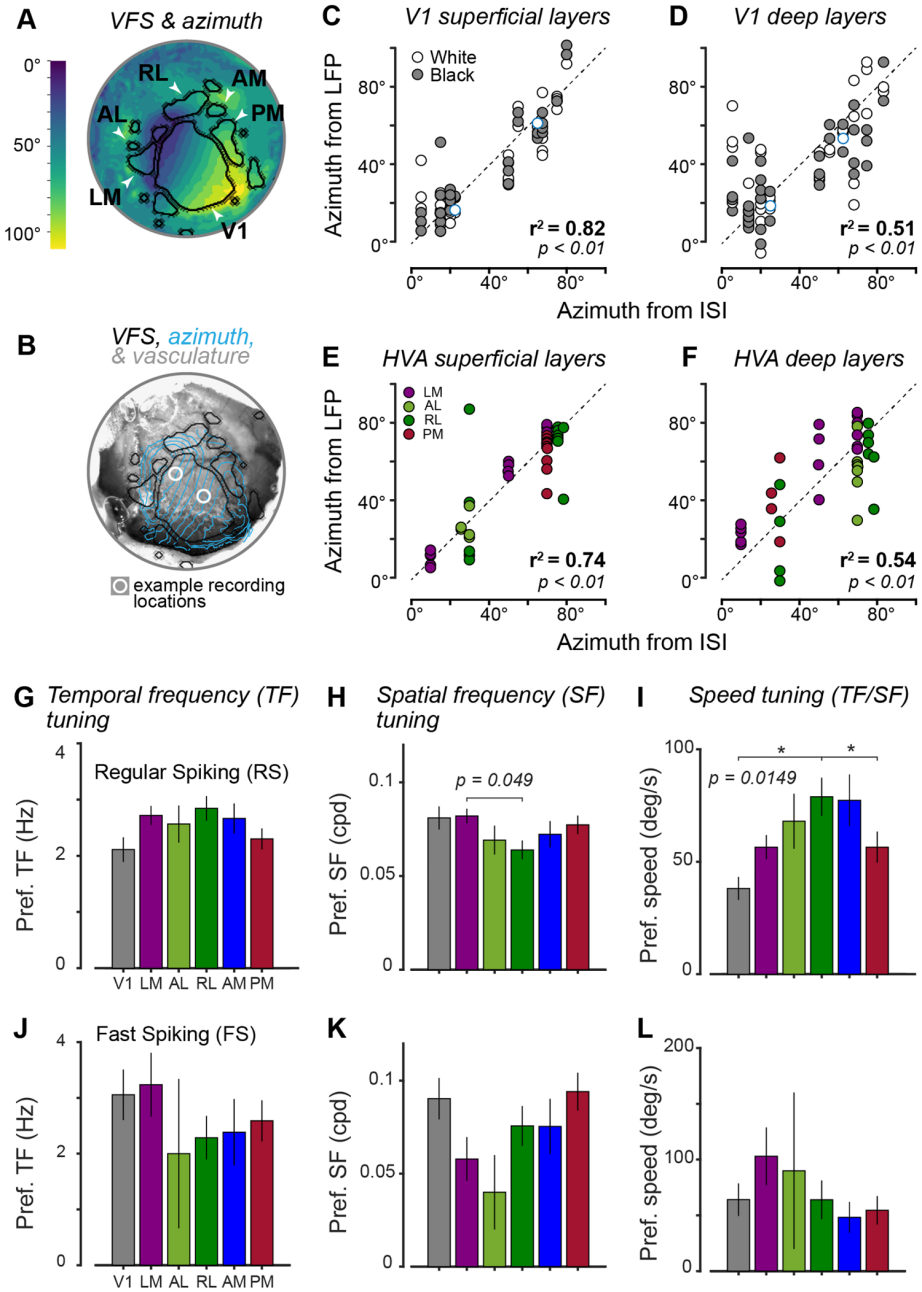
Validation of ISI map retinotopy in V1 and HVAs with electrophysiology. (**A**) Example overlay of VFS and azimuth retinotopy. Note HVAs show distinct retinotopic coverage (e.g., LM versus PM). (**B**) Overlay of VFS (black) and azimuth retinotopic map contours (blue, 10° increments) aligned with vasculature. White circles: sites and average size of craniotomies after cranial window removal and alignment to vasculature. Location of craniotomies is used to determine expected azimuth RF location within V1 or HVAs. Data in (**A**,**B**) from Mouse 1 (Fig. S3). (**C**) Correlation between expected ISI azimuth coordinates (abscissa) versus observed RF location from local field potential (LFP) responses (ordinate) in superficial layers of V1 (n = 6 mice, 22 recording sessions). Error: 8.2° ± 7.6° (mean ± SD). Overall r^2^ = 0.82, *p* = *3.8e*^*−31*^; Black stimulus r^2^ = 0.84, *p* = *5.9e*^*−17*^; White stimulus r^2^ = 0.80, *p* = *2.4e*^*−15*^. no significant difference between expected and observed (*p* = *0.39*) and white and black not significantly different (*p* = *0.46,* Wilcoxon signed rank test). Blue circles indicate recordings from mouse and sites in (**B**). Data from mice 1,2,3,5,6,7 in Supplemental Fig. S3. (**D**) As (**C**), for deep V1 layers. Error: 13.4° ± 13.1° (mean ± SD). Overall r^2^ = 0.51, *p* = *5.2e*^*−14*^; Black stimulus r^2^ = 0.61, *p* = *2.1e*^*−9*^; White stimulus r^2^ = 0.43, *p* = *3.0e*^*−6*^. No significant difference between expected and observed (*p* = *0.07*). (**E**,**F**) Like (**C**,**D**) for higher visual areas AL, LM, PM, and RL (n = 4 mice; 15 recording sessions). Superficial layers of HVAs show greater correlation to ISI coordinates (r^2^ = 0.74, *p* = *2.6e*^*−13*^) than deep layers (r^2^ = 0.54, *p* = *2.9e*^*−8*^). Data from mice 1, 2, 8, 9 in Supplemental Fig. S3 are shown here. (**G**–**I)** Regular spiking (RS) neuron temporal frequency, spatial frequency, and speed tuning in V1 (n = 82 neurons) and HVAs (LM: n = 181; AL: n = 46; RL: n = 108; AM: n = 67; PM: n = 125). Data from mice 1, 2, 9 (Fig. S3). No significant effect of area for TF tuning (*p* = *0.17*). Significant main effect of area on SF tuning (*p* = *0.049)*, with LM preferring higher SFs than RL. Significant main effect of area on speed tuning (*p* = *0.0148*), with RL preferring higher speed stimuli than V1 and PM. Kruskal–Wallis tests with Bonferroni correction for all comparisons. (**J–L**) Same sessions as (**G**–**I**), for fast spiking (FS) neurons in V1 (n = 27 neurons) and HVAs (LM: n = 19; AL: n = 4; RL: n = 28; AM: n = 13; PM: n = 34). No significant effect of area on FS tuning properties (TF: *p* = *0.615*; SF: *p* = *0.057*; Speed: *p* = *0.624*).

We next used ground-truth extracellular electrophysiological recordings to validate retinotopy estimated in the ISI maps of V1 and HVAs. The cranial window was removed by carefully drilling away the Metabond surrounding the coverslip, then detaching it and the supporting Vetbond. Once the window was removed for electrophysiology, we were able to visualize vascular landmarks and use these to target multiple craniotomies to primary and higher visual areas for multiple days in a row (n = 10 mice, 4–14 recording days), with high single unit yield and low noise signals. Craniotomies were performed in the primary visual cortex (V1), and higher visual areas: AL, LM, AM, PM, and RL (Fig. 4A). We aligned and overlaid the azimuth and VFS maps on the vasculature image to target specific retinotopic regions of the visual areas (Fig. 4B). For instance, LM and RL were expected to respond to a wide extent of azimuthal visual space (~ 0° to 100°). However, PM was expected to be responsive to stimuli in a more restricted azimuthal portion of monocular visual space (45° to 80°), consistent with previous findings^8^.

Extracellular recordings were performed to determine the preferred stimulus position in both azimuth and elevation for local field potential (LFP) responses^24,25^. Laminar LFP responses were separated into superficial and deep cortical layers (see “Methods” section). In V1, we found a high correlation between retinotopic coordinates estimated from ISI and electrophysiology in superficial cortical layers (Fig. 4C; r^2^ = 0.82, *p* = *3.8e*^*−31*^; average error: 8.2° ± 7.6°, n = 22 recordings in 6 mice). During these same recordings, deep layer LFPs also showed significant but weaker correlation to ISI coordinates (Fig. 4D; r^2^ = 0.51, *p* = *5.2e*^*−14*^) and with greater average error (13.4° ± 13.1°). Nevertheless, the error between ISI versus LFP retinotopic coordinates was not statistically significant in either superficial (*p* = *0.39*) or deep layers (*p* = *0.065*, Wilcoxon signed rank tests). Due to experimental considerations and time constraints during electrophysiological recordings, we prioritized verification of azimuth retinotopy in V1 and HVAs (discussed next). However, in a subset of V1 recordings (n = 3 mice, 13 recordings), we also measured elevation retinotopy. We again found low error between ISI maps and V1 LFP elevation retinotopy in superficial layers (3.5 ± 2.6°), compared to deep layers (6.9 ± 2.8°; mean ± SD). Accounting for error in both azimuth and elevation, the total estimated error in Euclidean space was 8.9 ± 8.0° (mean ± SD) in superficial layers and 15.1 ± 13.4° in deep layers of V1 (n = 35 recordings).

The correlation between ISI and LFP retinotopy in HVAs was also significant in all recordings, and greater in superficial layers (Fig. 4E; r^2^ = 0.74, *p* = *2.6e*^*−13*^) versus deep layers (Fig. 4F; r^2^ = 0.54, *p* = *2.9e*^*−8*^). In HVAs, only area LM exhibited a statistically significant difference between ISI and LFP retinotopy in both superficial (*p* = *0.023*) and deep cortical layers (*p* = *0.0027*; Wilcoxon signed rank tests). No significant differences were found between retinotopy estimated from ISI versus LFP in superficial or deep layers in areas AL (superficial, *p* = *0.625*; deep, *p* = *0.375*), PM (superficial, *p* = *0.195*; deep, *p* = *0.0781*), and RL (superficial, *p* = *0.583*; deep, *p* = *0.1721*).

Finally, in a subset of experiments, we measured functional properties of single neurons in HVAs and compared these to benchmark literature. We isolated single neurons in V1 and 5 of the HVAs targeted by ISI (LM, AL, RL, AM, PM) with silicon probe recordings in awake mice (n = 734 neurons, 3 mice, 29 recordings). Spatial and temporal frequency (SF and TF) tuning in regular spiking (RS) putative excitatory neurons in HVAs targeted by ISI showed broad consistency with prior reports, although small samples sizes largely precluded robust findings of statistical significance across all areas. We found a significant main effect of visual area on SF tuning (*p* = *0.049)*, with LM preferring higher SFs than RL (Fig. 4I; Kruskal–Wallis tests with Bonferroni correction for all comparisons). There was no significant main effect of visual area on TF tuning (*p* = *0.17*). However, when examining preferred stimulus speed (the ratio of preferred TF to preferred SF), we found a significant main effect of visual area on speed tuning (*p* = *0.0148*), with RL preferring higher speed stimuli than V1 and PM, broadly consistent with prior findings in V1, RL, and PM^26^. Again, these findings should be interpreted cautiously given low sample sizes relative to prior studies that sampled thousands of neurons^26^. Other differences between our and prior results include measuring neural activity across all layers, measuring spikes with silicon probes, not imposing single neuron inclusion criteria^27^, and measuring in awake stationary mice (rather than prior studies measuring Ca^2+^ responses from highly responsive neurons only in L2/3 of anesthetized^26^ or running^28^ mice). Overall, our findings suggest RS neurons prefer different stimulus speeds in V1, RL, and PM, a topic for future electrophysiological studies. These results, alongside verifications of expected retinotopy in HVAs (Fig. 4E,F), provide further evidence of the viability of our system for ISI targeting of HVAs with subsequent multi-site electrophysiology in awake mice.

## Discussion

Here, we validated the performance of an intrinsic signal optical imaging (ISI) system optimized for intact skull imaging in the adult mouse visual system. We characterized the quality, resolution, and trial dependence of retinotopic maps in multiple visual cortical areas, and then validated these with targeted electrophysiological measurements of retinotopy and functional properties. Our intact skull imaging in adult mice matches well-established benchmarks for thinned skull or excised skull cranial window preps^2,5,7,29^, but provides a specific advantage for investigators wishing to identify retinotopic maps and then perform subsequent visually targeted multi-site electrophysiology, perhaps weeks or months later (e.g. in mice undergoing training in behavioral tasks). Maintaining skull integrity over weeks to months ensures optimum mechanical stability and physiological conditions during sensitive electrophysiological recordings, particularly high-density multi-site silicon probe or patch clamp recordings^13,14^. We also improved quality control and alignment algorithms so that multiple imaging sessions within and across days can be readily combined; this enables multiple short-duration imaging sessions to be aggregated to resolve small or low-signal HVAs, rather than necessitating a single long-duration imaging session. We provide all necessary details to replicate these procedures and have made all code and methods available for those wishing to implement this minimally invasive ISI imaging that is readily combined with subsequent targeted, multi-site electrophysiology.

Development of this system also allowed us to provide quantification for the relationship between imaging duration and the resolvability of retinotopic maps in V1 and HVAs. Unlike previous research that has thoroughly inspected the variations in location and size of V1 and HVAs using excised skull cranial windows^6^, our study sought to determine the minimum number of trials (and thus minimum duration of anesthesia) needed to accurately resolve V1 and multiple HVAs through an intact skull cranial window. We found that displaying the visual stimulus for 50 to 60 trials in forward and reverse directions (~ 65 to 75 min) is sufficient to generate high-quality retinotopic maps that define the extent of V1 and 2 commonly investigated HVAs (PM, RL). ROC analysis revealed that ~ 60 visual stimulus trials identify retinotopy and delimit borders for V1 and these HVAs with > 75% accuracy, with ~ 90 trials needed to readily identify V1, LM, RL, AL, AM, PM (Fig. 2C). Somewhat surprisingly, resolving the full extent of area LM required the most trials. This could be because the full extent of LM can only be defined once V1 and AL (sign negative areas) and RL and Area P (sign positive) are also resolvable.

Comparison of our maps to prior studies reveals additional factors to consider for the resolvability of HVAs. First, imaging through thinned or excised skull for longer periods of time will yield better identification of areas beyond the main group of lateral (LM, RL, AL) and medial (AM, PM) HVAs, an important consideration for targeted investigation of such areas^30^; Second, VFS maps constructed from widefield GCaMP6 fluorescence—a direct neuronal signal—provide higher resolvability and faster identification of HVAs than ISI maps^2,6^, providing advantages over hemodynamic imaging but limiting experiments to transgenic mice expressing calcium indicators. Other benchmark studies of hemodynamic ISI mapping that use both intact skull and transcranial imaging show high resolution maps^8,9^, but the exact skull preparation and imaging durations generating the exemplar maps are not specified and thus difficult to directly compare with ours; one of these prior studies acquired maps with very long periods of imaging (up to 6 h) in acute tracheostomized subjects. Although many prior studies have used intact skull transcranial preps for acute ISI in juvenile^29^ or adult mice^15,31,32^, our study specifically developed a chronic transcranial window for adult mice that (1) generates high quality ISI maps of V1 and HVAs while minimizing the extent of anesthetized imaging (2) allows visualization, monitoring, and maintenance of cranial and cortical health for weeks to months, and (3) facilitates visually targeted multi-site electrophysiology from V1 and HVAs within subjects, discussed next.

Validation of this system with electrophysiology allowed us to quantify the relationship between retinotopy inferred by ISI maps versus retinotopy measured from neural activity across cortical layers. We confirmed that ISI retinotopy showed significant correlation with LFP retinotopy in V1 and multiple HVAs; furthermore, ISI retinotopy corresponded most closely with neural activity acquired from the superficial layers of cortex, with an error (± 8.2°) comparable to the width of the visual stimulus presented during electrophysiology experiments (9°). This error is much smaller than the average receptive field size of V1 excitatory neurons (between 15°–30°)^33,34^. ISI also showed significant correlation with retinotopy in deeper layers, but with greater error (± 13.4°), consistent with prior observations that deep layer V1 neurons show larger RFs and greater retinotopic scatter^33,34^. These findings carry some limitations. First, we did not systematically verify that each electrode penetration was completely perpendicular to the cortical surface, which could contribute to greater variability in our deeper layer measurements; second, electrophysiology was performed in both anesthetized and awake mice, which could contribute to greater variability. Nevertheless, to the best of our knowledge, our study provides the first error estimates for retinotopy inferred from ISI maps versus laminar-specific neural activity across multiple mouse visual cortical areas. In all cases, measures of retinotopy inferred from transcranial ISI showed significant correlation with direct electrophysiological measures in V1 and HVAs. These findings also provide considerations for future studies of laminar-specific neural activity underlying ISI signals. Finally, functional visual properties of single neurons in V1 and HVAs in our ISI targeted recordings showed some consistency with benchmark literature^26^, providing a second independent electrophysiological metric of ISI map fidelity.

Our system and protocol were optimized for both novice and experienced users, yet some limitations remain. First, the quality of signals and retinotopic maps depends critically upon the clarity and stability of the window; this requires some skill and experience for success but is no more difficult than many other in vivo mouse procedures requiring careful execution (e.g., headplate implantation, cannulation, stereotaxic injections). Second, the system and protocol has only been optimized with a single brand of CMOS camera and frame grabber, although the code could be readily adaptable to other hardware, including sCMOS cameras. Third, the system and protocol still require human intervention (e.g., adjustment of camera focus, light intensity, or anesthesia level), but this is described here step-by-step. Fourth, our quantitative assessment of differences between ISI vs neural signals mainly considered azimuthal retinotopy, but future studies could consider other aspects of visual selectivity. Finally, our methods for transcranial ISI and subsequent electrophysiology seem readily testable in other sensory systems^35,36^.

In summary, this system provides a way for investigators of the mouse visual system to pair well-established hemodynamic mapping of visual cortical brain activity^9,37^, with subsequent long-term, stable, retinotopically targeted neural recordings across multiple cortical visual areas. We have characterized system performance for minimally invasive intact skull cranial windows and measured the minimum amount of anesthetized imaging data required to infer reliable retinotopic maps of V1 and several HVAs. Minimizing the need for bone thinning, bone or dura removal, or recovery from long bouts of anesthesia is particularly advantageous to investigators using mice for experiments requiring manipulations lasting weeks to months (e.g., behavioral tasks, plasticity studies, recovery of visual function, studies of aging). Lastly, the minimally invasive requirements for high resolution ISI mapping shown here may be more amenable for sensitive or costly transgenic strains, therefore expanding capabilities for experiments that require precise targeting of cortical visual areas to study mouse models of neurological disorders.

## Methods

All experiments were approved by the Georgia Institute of Technology Institutional Animal Care and Use Committee (IACUC) and conform to guidelines established by the National Institutes of Health. All methods were performed in accordance with the relevant guidelines and regulations. The study design did not use treatments necessitating blinding, or comparison of experimental versus control groups. All descriptions of experimental procedures, sample sizes, data analysis, resampling methods, statistical comparisons, and outcome measures are consistent with the ARRIVE guidelines 2.0.

### ISI hardware and software

All relevant software packages and toolboxes for intrinsic signal optical imaging are included in the ISI package available in the lab’s public code repository (https://github.com/haiderlab/ISI). The initial list of components and software was obtained from a previously published protocol^5^. It was then adjusted to meet logistical requirements and conditions in our lab (e.g., updated software for acquisition and processing, updated DAQ and camera interface, updated acquisition of frame timestamps, expansion of visual display from 1 to 2 monitors, etc.). Briefly, our ISI system was composed of a primary and secondary computer (control center and stimulus display), a light supply, a photodiode for temporal alignment, and a tandem-lens macroscope for image acquisition (Fig. 1A). MATLAB R2018b was used to develop, optimize, and run all software, and process and analyze all data.

The current system codebase has been improved in several ways relative to prior open-source ISI systems and extended to facilitate integration with electrophysiology experiments. First, we have adapted the original source code^5^ (MATLAB v. 2008) to function with modern versions of MATLAB that use different DAQ interfacing (validated here from MATLAB v. 2018 onward). Second, we have expanded the visual stimulus display to include two monitors placed further away from the mouse, enabling placement of equipment necessary for neural recordings in the same set-ups (micromanipulators, lick detectors, recording accessories) while still permitting stimulation and mapping of large regions of visual space. Third, we have removed system dependency on the Matrox Imaging Library with custom C^++^ code. Fourth, we deployed a method to trigger frame captures independently from visual display draw, to timestamp these frames using the system clock, and then to align acquired frames to the start and stop of visual stimuli and monitor frame flips using a simultaneously acquired photodiode signal. Fifth, we have created the ability for users to quickly overlay and register visible light images of the skull and craniotomies with user-defined fiduciary landmarks to overlay craniotomies on retinotopic maps. Lastly, we have provided a simple GUI for system control for novice users, with an option for greater control by expert users.

To image the cortex, a wide-field camera, controlled by a frame grabber (Matrox Radient eV; Matrox), with lenses (Lens 1: Nikon—AI-S FX Nikkor 50 mm f/1.2 manual focus lens; Lens 2: Nikon—Ai 85 mm f/2 manual focus lens) in tandem configuration was positioned above a transcranial window. Frames were captured while visual stimuli (see “Visual stimuli” below for details) were presented on two screens covering the binocular and monocular visual space (Fig. 1A). Custom-built software was used to interface all hardware components, provide feedback during hemodynamic imaging, conduct in-depth post-recording analysis, and align ground-truth electrophysiology craniotomies to retinotopic maps.

The general steps and code are outlined in Table 1.

Common Troubleshooting steps are outlined in Table 2.

### Surgical procedures

All procedures were approved by the Georgia Institute of Technology Institutional Animal Care and Use Committee (IACUC). We present data from the same n = 10 implanted mice throughout the study (Fig. S3), including 5 C57BL/6J (Mice 1, 2, 6, 7, 8), 3 B6PV^Cre^ x Ai32 (Mice 3, 9, 10), and 1 Sst-IRES-cre x Ai32 mouse (Mouse 5). We did not observe obvious differences in signal quality in mice expressing a fluorescent protein (Fig. S3). Additional imaging data from 1 CNTNAP2^−/−^ KO mouse^24^ (Mouse 4) is shown for repeatability analysis, with no contribution to electrophysiological analysis. Mouse 10 also contributed only imaging data (Fig. S4B), so n = 8 mice were used for both imaging and electrophysiology (Mice 1–3, 5–9). All mice were male and 4–12 weeks old at time of implant. Detailed procedures for head-plate implantation have been described elsewhere^25^. Briefly, mice were anesthetized with isoflurane (3% induction, 1–2% maintenance), body temperature was kept at ~ 37 °C using a heating pad, and the eyes were protected with veterinary ophthalmic ointment (Puralube). The skin was removed, and the fascia and periosteum overlying the skull were carefully resected and removed with a cotton bud and/or scalpel blade (no. 11) under physiological saline solution, avoiding scratches or bleeding of the cranial surface. No removal or thinning of the skull was performed following this step. Once dural and cortical vasculature was cleanly and clearly visible under saline, the skull was allowed to air dry thoroughly. A custom-built stainless steel head post with a recording chamber (11 mm inner diameter) was lightly affixed to the skull using veterinary adhesive (Vetbond) (Supplementary Fig. S2). Following headplate fixation, a glass coverslip (5 mm diameter, #1 thickness ~ 0.15 mm, Warner CS-5R) was centred over the representation of V1 and HVAs (centre of window at ~ 2.4 mm lateral to midline and ~ 2.4 mm anterior to lambda) and bonded to the skull using Vetbond (Supplementary Fig. S2). The layer of Vetbond between the glass window and skull was allowed to fully dry (45–75 min), leaving a fully transparent transcranial view of cortical surface vasculature. The edges of the cranial window were then sealed with dental polymer (Metabond), and the headplate was fully bonded to the skull. Mice were individually housed and monitored for full recovery for at least 3 days before imaging.

### Imaging procedures

Mice were anesthetized with isoflurane (3% induction), given a sedative via intraperitoneal injection (Chlorprothixene, 10^−5^ mg/kg), and placed on a heating pad to maintain body temperature (~ 37 °C). 3-mm contact lenses (Ocuscience) were inserted to prevent dehydration of the eyes and maintain ocular clarity during imaging sessions. During imaging anesthesia was lowered to 0.5–0.75%. The cortex was illuminated using fiber optic guides and a high intensity tungsten halogen lamp (Illumination technologies 3900E, 9596A lamp) that emits high intensity light across a broad wavelength spectrum (Fig. S2B). We placed a bandpass green filter (450–600 nm; Illumination Tech P/N 9542) between the light source and fiber optic guides to better isolate signals from vasculature, or used a longpass red filter (> 610 nm; Illumination Tech P/N 9541) to better isolate changes in blood oxygenation (see discussion below). A filter wheel (Thorlabs LCFW5) with bandpass emission filters (Edmund Optics) was installed between the macroscope lenses to capture reflected photons from green (525 ± 25 nm) or red (700 ± 10 nm) imaging, as in prior studies^5,29,38^. A CMOS camera (Falcon2; Teledyne DALSA) acquired images of the cortex at a frame rate of 10 Hz. We choose to use a CMOS camera to maintain system consistency with prior work, but the integration of sCMOS cameras is an avenue for future system optimization.

The general procedure for finding the optimal focal plane consists of the following steps. First, the mouse is positioned and secured on the recording rig, and the camera is placed above the cranial window, and brought to maximal height. Green light and filters are enabled, and the height of the camera is decreased just until blood vessels are clearly visible. Then, using a calibrated scale attached to the camera, we lower the focal plane by another 0.5 mm so that vasculature appears slightly blurry. We then proceed to perform a test experiment (with green light). If the test fails to show adequate signal, the experimenter can perform a series of interventions, including re-adjustment of the camera focus. The best rule of thumb is to re-focus on the vasculature then decrease the focal plane by greater than 0.5 mm, and proceed with another test acquisition. This step can be iterated up to 1 mm below the vasculature surface, but no deeper. If the experimenter is not able to find a clearly optimal focal plane, imaging just below the level of vasculature is usually adequate for subsequent red light imaging.

The imaging wavelengths of our system are based on prior established studies of intrinsic signal imaging in mice^5,7,8,26,39^. Wavelengths 510–590 nm (green) primarily isolate changes in blood volume that result from a combination of vasculature dilation, capillary blood recruitment and cortical activity^21^. Wavelengths > 600 nm (red) permit better isolation of the oxymetric component of the hemodynamic signal, due to differences in sensitivity of light absorption for deoxygenated versus oxygenated hemoglobin. Imaging the brain at these wavelengths produces ISI maps that are more spatially correlated to the underlying neuronal response than maps produced from shorter wavelengths that primarily reflect widespread blood volume changes^21,40^. Although some studies suggest optimal signal to noise ratio for oxymetric signals at 610–630 nm^41^, at ~ 700 nm there is a substantial increase in the relative absorption differences between deoxygenated versus oxygenated hemoglobin^42^, which further accentuates detection of active metabolic changes due to neural activity. These considerations motivate the wavelength choices in our system, as well as in recent studies that use identical wavelengths for cortical ISI in mice^5,29,38^.

### Visual stimulus for ISI

Mice were positioned in front of two computer monitors that were at right angles from one another (Fig. 1A). Mice were facing the center of one monitor covering the binocular visual field, and 90° from the center of the second monitor covering the monocular visual field. Stimuli were presented on linearized LCD monitors (60 or 80 Hz refresh rate; Dell U2419H with maximum brightness of 250 cd * m^−2^; mean [black, grey, white] screen luminance during recordings of [0, 112, and 238] cd * m^−2^). The stimulus was a 20° wide with black and white full contrast reversing checkerboard (6 Hz), drifting at 0.055 Hz across the visual field on a black background. The horizontal drifting stimulus was corrected for spherical visual coordinates^26^.Our decision to use a continuous reversing checkerboard drifting bar to drive the cortex was to maintain consistency with stimuli used to generate ISI maps in prior studies^5,9,26,43^.

Screens were positioned ~ 19 cm away from the eyes, and the mouse was vertically positioned at the midpoint of the screens (~ 17 cm above air table) (Fig. 1B). The vertical and the horizontal planes through the eyes were used to define the origin of azimuth and elevation visual coordinates (0° directly in front of the mouse). Visual stimulus presentation consisted of a checkerboard drifting in any of the four cardinal directions (Nasal-Temporal, Temporal-Nasal, Superior-Inferior, Inferior-Superior) for 18 s. Each block consisted of 10 unidirectional sweeps lasting a total of 180 s. Imaging sessions were comprised of multiple blocks. Pairs of stimulus sweeps (in opposite directions) generally defined a single trial for absolute phase map construction (see below, “Hemodynamic correction”).

### Images acquisition, processing, and quality control

#### Signal quality check with green light imaging

At the beginning of each experiment, a short test acquisition (Duration: 3–5 min) was performed in-focus with the surface vasculature with green light (λ 450–600 nm) and a 525 nm bandpass filter (see “Imaging procedures” section). This allowed experimenters to rapidly assess coarse visually evoked hemodynamic signals and perform any adjustments before further acquisition (Tables 1 and 2). Common adjustments included adjusting lighting position, intensity, and depth of focus. Adjustments and rapid test acquisitions were performed repeatedly until appropriate signals were detected (Table 1; Fig. 1C). Following this verification step, the camera was focused below the cortical surface (~ 100–500 μm below the brain surface), and long imaging sessions (Duration: 1–2.5 h) were performed with red light (λ > 610 nm) and 700 nm bandpass filter to isolate changes in deoxyhemoglobin (HbR) concentrations across the visual cortex. Mice were subjected to one imaging session per day. Each imaging session was comprised of multiple blocks of visual stimulus presentation in multiple directions (see “Visual stimulus for ISI” section). Images were collected at 10 Hz (180 frames per trial).

#### Hemodynamic delay correction

The slow temporal sampling of ISI signals (~ 10 Hz) is adequate to capture hemodynamic responses because they evolve slowly compared to the time course of neuronal activity^44,45^. Previous research has shown that the detection of adequate hemodynamic responses during anesthesia necessitates visual stimuli with periods > 10 s^9^. Unlike electrical activity, blood perfusion-related responses are usually delayed by at least 1–6 s from the onset of a stimulus^7,40^. This hemodynamic shift can be corrected by recording responses in one cardinal direction (forward motion) and its reverse (backward motion), then subtracting them to create an absolute response map^9^. Thus, intrinsic signals are recorded with repeated continuous visual stimuli sweeping across the screens in directional pairs: Nasal-Temporal and Temporal-Nasal for mapping azimuth, or Superior-Inferior and Inferior-Superior for mapping elevation. Here, retinotopic maps (azimuth and elevation) were produced from a minimum of 5 blocks/session (50 trials) in both the forward (Azimuth: Nasal–Temporal; Elevation: Inferior–Superior) and backward (Azimuth: Temporal–Nasal; Elevation: Superior–Inferior) directions. A maximum of 10 blocks (100 trials) per imaging session were acquired. One directional pair constitutes the definition of a trial for analysis of absolute phase maps of azimuth and elevation (Fig. 3A,B), and then matched directional pairs (one pair for azimuth maps, one for elevation maps) constitute a trial for a VFS map (since VFS maps are necessarily computed from the angular difference between the azimuth and elevation absolute phase maps).

Images were processed following previously published methods^2,8,46^, with additional adjustments. First, images from a block were aligned, cropped, and resized to the vasculature image acquired at the start of each session using green light (Supplemental Fig. S1A–C). Then, the baseline signal (average of frames acquired during the first 5 s when no visual stimulus was presented) was subtracted from each frame to retrieve the change in light absorbance. This was done using the modified Beer-Lambert law^46^:

$$\Delta A= \varepsilon l\Delta C={log}_{10}\left(\frac{{I}_{a}}{{I}_{0}}\right)$$

where the change in light absorbance is $\Delta A$, molar absorptivity is $\varepsilon$, path length is $l$, molar concentration change compared to baseline measurement is $\Delta C$, post stimuli light intensity is ${I}_{a}$, and baseline light intensity is ${I}_{0}$.

#### Fourier analysis of response phase maps

A Discrete Fourier Transform (DFT) was used to extract each pixel response at the frequency of the visual stimulus to create periodic phase maps^2^. Phase maps depicting the average change during each trial within a block were constructed (i.e., 10 single trial phase maps per block). A quality criterion was then applied to only select single trial phase maps that exceeded a normalized variance of 0.6 (calculated as variance of pixel intensity from the mean in absolute phase maps). Single trial phase maps that exceeded the normalized variance threshold were combined to create the average block phase map. To overcome the hemodynamic delay following the presentation of a stimulus, absolute phase maps were constructed by subtracting the block averaged phase map of the backward motion (e.g., Temporal–Nasal) from the block averaged phase map for forward motion (e.g., Nasal–Temporal). These absolute phase maps were then translated to the spatial location of the visual stimulus to create azimuth or elevation retinotopic maps (Fig. 2A,B). Multiple block averaged absolute phase maps were combined to constitute the imaging session phase map. At each pixel, we computed the sine angle difference between azimuth and elevation maps to create a visual field sign (VFS) map (Fig. 2C). VFS maps use features of retinotopic gradients (e.g. regions where retinotopic preferences reverse polarity) to define the size and boundaries of the primary (V1) and higher visual areas (HVAs). Here, the direction of each pixel’s retinotopic gradient is represented as a value ranging from sign negative (− 1) to sign positive (1). Additionally, the software aligns and overlays the VFS map on the vasculature and retinotopic contours (Fig. 2D,E).

### Alignment and overlay of retinotopic maps to vasculature

Contour maps in azimuth and elevation were automatically aligned to the reference vasculature image, obtained with green light during post-imaging analysis (Supplemental Fig. S1A–C). First, images were cropped to only preserve the region of interest from the retinotopic and vasculature images. Then, the images were resized before being overlaid (Fig. 2D,E). After the cranial window was removed for electrophysiological recordings, a reference image showing the site(s) of craniotomies, vasculature, and fiduciary landmarks in the chamber was used to semi-automatically align the retinotopic maps using custom functions (“Align.m”). These are rigid transformations (X–Y translations) with no rotation or warping to account for different focal planes. The investigator selects common fiduciary features in both retinotopic map + vasculature image as well as craniotomies + vasculature image to initiate the alignment.

### Neural recordings and analysis of retinotopy

The detailed steps for laminar local field potential recordings have previously been published^25,47^. In short, small craniotomies (~ 100–500 μm) were made over V1 or HVAs using the ISI maps as reference. Recordings were made with multi-site silicon probes (Neuronexus) consisting of a single 32-channel shank spanning all layers of the cortex. Electrodes were advanced ~ 1000 μm below the cortical surface. The signals were acquired at 30 kHz (Blackrock Microsystems) and filtered at 0.3–300 Hz to acquire the LFP signal. To measure the preferred retinotopic locations for neural responses, 100% contrast vertical white or black bars (width: 9°, duration: 0.1 s, inter-stimulus interval: 0.3 s) were presented in random locations spanning the binocular and monocular visual field (~ − 60° to + 150° in azimuth) on grey linearized LCD monitors (see specs in “Visual Stimuli for ISI"). LFP recordings were performed in anesthetized (n = 5) and awake (n = 5) mice as detailed previously^24^. We observed no major differences in retinotopic correspondence of ISI versus LFP across anesthetized (V1, RL) and awake (V1, RL, LM, PM, AL) recordings, so these were combined (Fig. 4).

### Analysis of retinotopic maps in V1 and HVA

In order to create contour maps, azimuth and elevation retinotopic maps were rounded to the nearest 10°, with each contour section representing the cortical area that responds to that visual stimulus spatial location. Data from all imaging sessions (i.e., all ISI sessions across multiple days) were then used to create randomly drawn subsamples of specific sizes from the total dataset. Resampling of data was done with or without replacement, with no obvious differences between methods (Fig. 3A). These subsampled maps of increasing data length were then compared to a reference map computed using all data collected from all ISI imaging sessions within a mouse. The similarity of subsampled and reference maps was evaluated by determining the centroid of each contour section for the subsampled and reference maps, then calculating the average Euclidean distance between the two (similar to prior approaches^6,8^). This enabled us to study the error in estimating cortical retinotopy as a function of number of trials. Subsampled and reference VFS maps were also created as above (Fig. S3), allowing us to plot the pixel intensity distribution within boundaries of identified cortical areas (Fig. 3C; Fig. S4A). We calculated receiver operating characteristic (ROC) curves of VFS pixel intensity using contour areas defined by the reference maps (“signal”) versus adjacent non-visual cortical (“noise”) regions outside of areas localized in the reference map (see Fig. 3D and Fig. S2D) to identify the number of samples needed to resolve V1 and HVAs.

### Analysis of retinotopic signals in ISI versus electrophysiology

We used retinotopic ISI maps to perform targeted craniotomies and neural recordings of specific retinotopic subregions within V1 and HVAs. The sites of craniotomies and azimuth retinotopic maps were aligned to identify expected ISI retinotopic coordinates and compare these to ground-truth electrophysiological measurements at these same sites (Fig. 4B). LFP responses were separated into cortical layers based on the earliest visual response latency, which typically corresponds to the input layer^1,24^. In V1, the channel with the lowest latency represents the middle of L4, which is about 200 µm in thickness. L4 was defined as the average of all channels within ± 100 microns of this site. The average of all channels above the upper boundary of L4 was termed superficial layers, whereas the average of all channels below the lower boundary of L4 was termed deep layers. Retinotopic preferences of the maximum LFP response (averaged within the superficial and deep layers) were then compared to the preferred locations predicted from the retinotopic ISI maps. Pearson correlations (r) were computed (MATLAB ‘corr’ function) between the ISI and LFP preferred locations to obtain r^2^ values. Additionally, the differences between ISI (expected) and LFP (observed) retinotopic coordinates provided an error estimate that could be compared across all experiments. We inspected if there was any dependence of expected versus observed coordinates upon the luminance polarity of the visual stimulus (black versus white, 100% contrast). Since there were no obvious differences, these are presented together (Fig. 4).

### Single neuron selectivity analysis in HVAs

We performed awake extracellular recordings as described above (“Neural recordings and analysis of retinotopy” section) across visual areas (V1, LM, AL, RL, AM, and PM). We isolated single neuron action potentials and separated these into regular spiking (RS) putative excitatory and fast spiking (FS) putative inhibitory neurons as in our prior studies^24,25,48^. Drifting gratings (σ = 10°, 100% contrast) were positioned within 10° degrees of the receptive field as determined by the LFP response online. Gratings varied in orientation and drift direction (0–360° at 45° intervals), spatial frequency (0.02, 0.04, 0.1, 0.16 cpd), and temporal frequency (0.5, 1, 2, 6 Hz). These parameters were chosen to match benchmark studies of neural selectivity in mouse higher visual areas^26,28^, and only recordings with receptive fields in the monocular visual fields were analyzed, again consistent with previous reports^26^. Single neuron responses were analyzed by first determining preferred direction (direction with the highest evoked firing rate) and then measuring the preferred spatial frequency and temporal frequency at the preferred direction (Fig. 4). Speed tuning was calculated as the preferred temporal frequency divided by the preferred spatial frequency.

## Supplementary Information

Supplementary Information.

## Data Availability

All ISI system code is deposited at https://github.com/haiderlab/ISI, and source data and analysis code to replicate the main results will be publicly available at DOI (https://doi.org/10.6084/m9.figshare.16200711) upon publication.
