# Supplementary material for: Optimizing intact skull intrinsic signal imaging for subsequent targeted electrophysiology across mouse visual cortex
Source: Sci Rep. 2022 Feb 8;12:2063. doi: 10.1038/s41598-022-05932-2 (PMC8826313; doi:10.1038/s41598-022-05932-2)
Supplement: Supplementary file 1 — Supplementary Information. [file 41598_2022_5932_MOESM1_ESM.pdf]

Supplementary Figures S1-S4

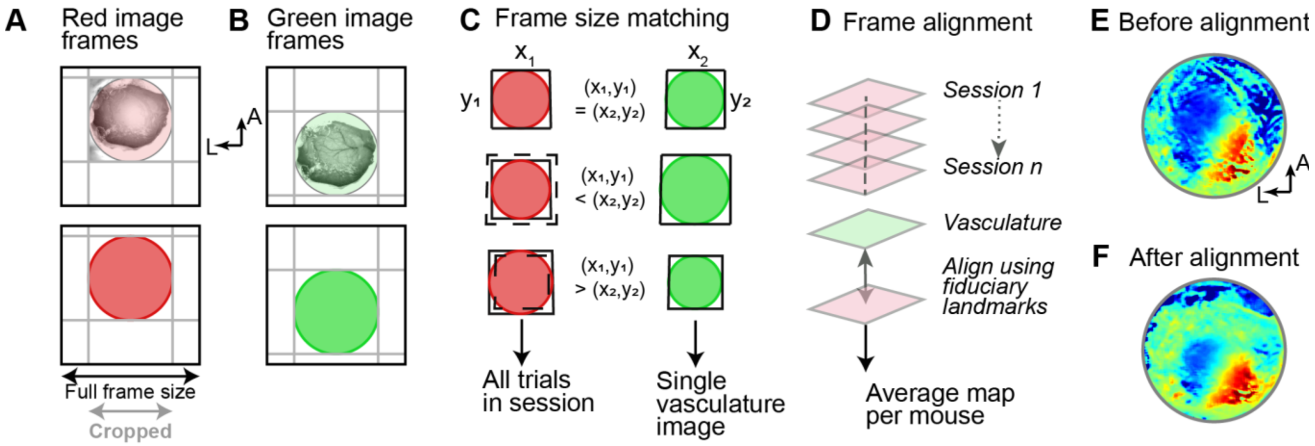

Supplemental Figure S1. Alignment of green and red light images.

A. Example frame during red light (>610 nm) imaging. Full frame size is larger than cranial window and cropped (grey lines) to delimit pixels restricted to the region of interest (ROI).

B. Same mouse and imaging session as A, under green light (~525 nm) imaging. Cranial window position within image frames can shift because of changes in camera position within day (e.g., after changing illumination sources), or across multiple days of imaging. Positional shift is exaggerated for illustration.

C. All red and green image frames are cropped to the ROI sized to the cranial window. Red cropped images are then matched to the dimensions of the reference vasculature image acquired under green light.

D. Image frames from red light imaging trials are collected per session. They are then processed (see panel A, B, C) and aligned to one vasculature image from that session before being averaged. Multiple session averages are stacked, re-aligned to one vasculature image, and averaged to make the averaged mouse retinotopic map.

E. Example session averaged azimuth map (same mouse as in A and B) before image cropping and alignment.

F. Same as E, after alignment procedure. Map shows greater continuity of retinotopy and clearer localization of extent and boundaries of V1. E and F from Mouse 1 (Fig. S3A).

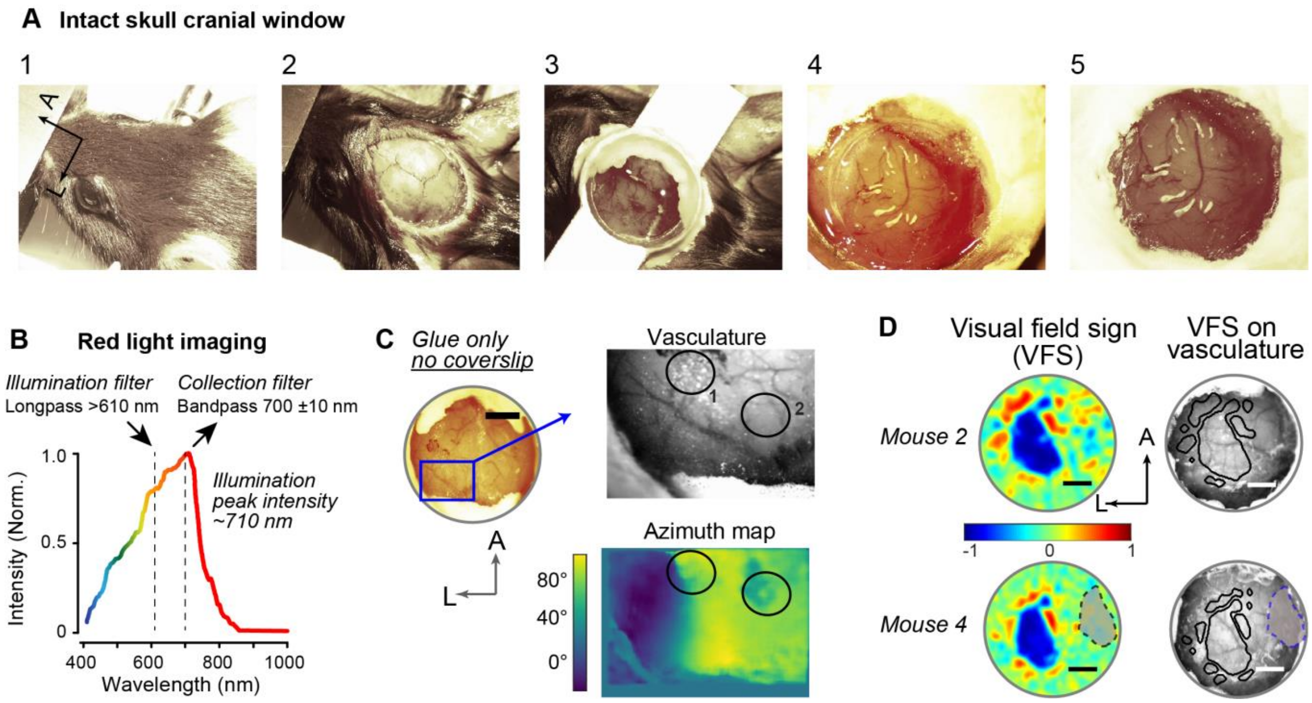

Supplemental Figure S2. Cranial window implant, red light imaging, and VFS map overlays on vasculature

A. Steps for implantation of intact skull cranial window. 1) Mouse is anesthetized and placed on surgery stage. 2) Skin, fascia, periosteum is carefully removed to expose cranium. 3) Head post (11-mm inner diameter recording chamber) is placed on skull and affixed with VetBond & Metabond. Once fully bonded, saline is added inside the recording chamber to assess transparency of the skull and visualization of vasculature. 4) Measurements from central and lambdoid sutures provide guidance in placement of glass coverslip over V1 and HVAs. Once the skull is dried, the glass coverslip (5-mm diameter) is bonded to the skull using VetBond. 5) After VetBond is fully polymerized under and around coverslip, window edges are further anchored with Metabond and attached to headplate. Any stray Metabond on the surface of the window is gently scraped away after the implant is fully bonded.

B. Spectral characteristics of ISI system during red light imaging. A longpass filter (>610 nm) is placed between the light source and fibre-optic guides placed above the cranial window. Note that illumination intensity peaks near 710 nm but intensity >50% of max spans a broad from 550 to 750 nm. A bandpass filter between macroscope lens and camera sensor limits collection of reflected photons to 700 ±10 nm. Illumination source intensity spectrum adapted from manufacturer technical documentation (Lamp 9596; https://illuminationtech.com/shop/ols/categories/tungston-halogen-light-source).

C. *Left*, example of cranial window prep using a different glue (CA Glue) with no coverslip. Scale bar is 2.75 mm. *Top right,* glue with no coverslip starts to show surface roughening (ROI 1) and areas of lowered reflectance (ROI 2). *Bottom right,* Azimuth map shows sudden discontinuities of retinotopic coordinates in ROI 1 and 2. Compare to smooth gradation of retinotopy below ROI 1.

D. Visual field sign (VFS) maps (left) and automated overlays of VFS on vasculature images (right) from two mice. These maps are used to target craniotomies using surface vasculature features. Azimuth and Elevation retinotopic maps of mouse 2 are shown on Fig. 2. Dashed grey area in Mouse 4 shows an example of non-visual area region of interest (ROI) used for V1 signal to noise analysis (Supplemental Fig. S4). Scale bar is 1 mm.

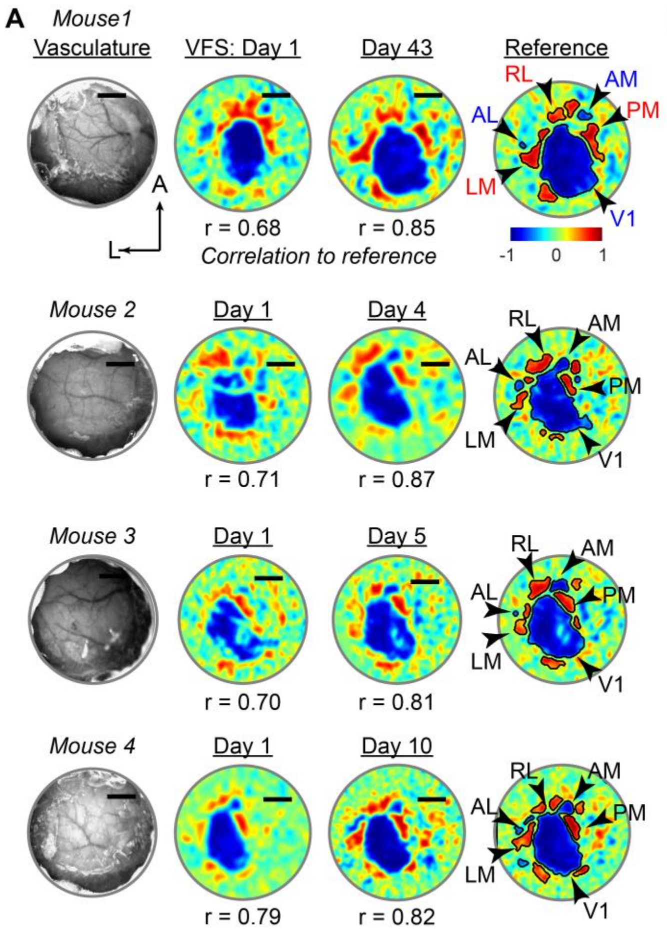

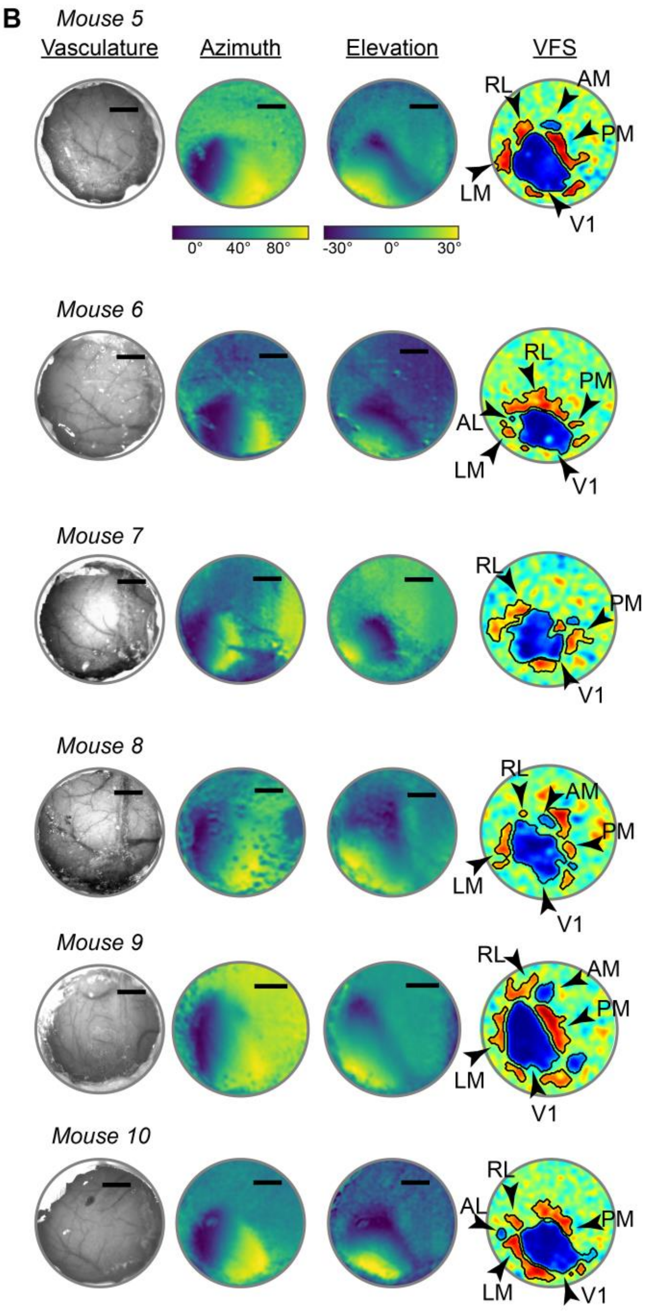

C

VISUAL AREA	*VISUAL FIELD SIGN*	*IDENTIFIED IN VFS MAPS (%)*
V1	-	*100%*
RL	+	*100%*
PM	+	*100%*
LM	+	*90%*
AM	-	*70%*
AL	-	*60%*

Supplemental Figure S3. Cranial windows and maps of VFS, azimuth, elevation for all mice in study

A. Intact skull cranial windows (left) and resulting visual field sign (VFS) maps on individual days (middle columns), relative to reference maps constructed from multiple days (right) in 4 mice. Pearson's correlation coefficient of each single day map relative to reference map is shown below each single day image. Note overall repeatability of VFS maps over time. Reference map shows automated contour delineation of sign negative areas V1, anterolateral (AL), and anteromedial (AM), and sign positive areas anteromedial (LM), rostrolateral (RL), and posteromedial (PM). Sign positive area P (posterior of V1) delineated but unlabelled. Scale bar = 1 mm. Azimuth and elevation maps for mice 1 and 2 shown in Fig. 2.

B. Additional examples of intact skull cranial windows (leftmost), Azimuth and Elevation retinotopic maps (middle two), and VFS maps (rightmost) in 6 more mice. Mouse 10 only contributed data to Fig. S4B.

C. Table of identification success for V1 and HVAs for all mice in A – B. Sign negative HVAs (AM, AL) showed lower resolvability than sign positive HVAs (RL, PM, LM).

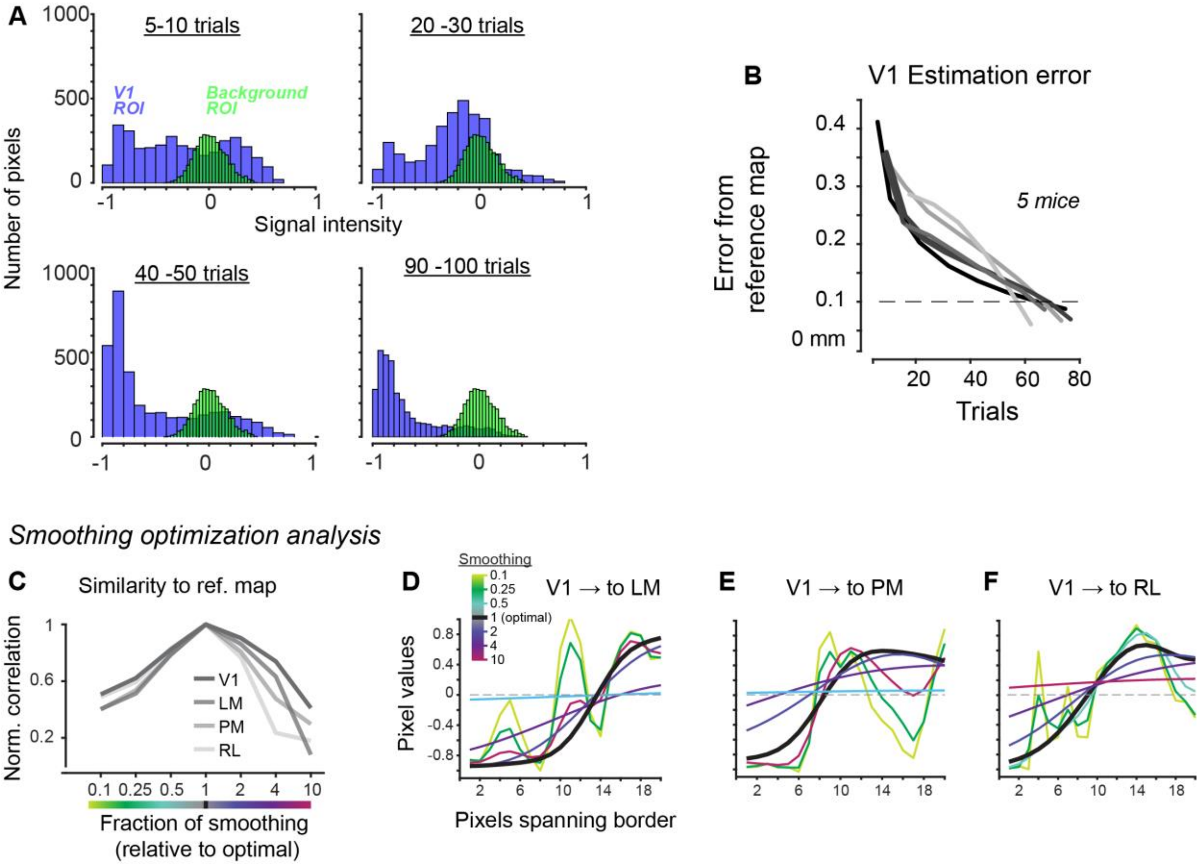

Supplemental Figure S4. V1 signal to noise analysis and error estimation in additional mice.

A. V1 signal to noise analysis. Distribution of V1 ROI pixel intensity (violet) and background ROI (green) as a function of trial number (V1 and background ROIs shown in Supplemental Fig. S2, Mouse 9). Data used for results in Fig.3 (Mouse 1). Clear separation of V1 ROI pixel intensity versus background noise distribution after 40-50 trials.

B. Error of estimated azimuth receptive field (RF) locations in V1 as a function of number of trials for additional mice. Each line represents observations from 1 mouse (n = 5; Mice 1, 2, 5, 6, 10 in Supplemental Fig. S3).

C-F. Smoothing optimization analysis. C. Similarity (correlation) of smoothed VFS maps to a fixed VFS reference map suffers for too much or too little smoothing from the optimal determined level (3-5 pixels; 18-30 microns). D-F. Similar detriments of non-optimal smoothing on smoothness of border transition from V1 to HVAs (LM, PM, RL). Here, the intensity of pixels in an array placed in VFS map between V1 (sign negative pixels) and 3 HVAs (LM, PM, RL; sign positive pixels) is analysed. Smooth and steep increase of pixel intensity from negative (V1) to zero (border), then to positive (HVA) is greatly compromised with smoothing parameters greater or lower than optimal. All data from Mouse 1 (Fig. S3A).
